# How to conduct a clinical audit and quality improvement project

**DOI:** 10.1097/IJ9.0000000000000024

**Published:** 2017-06-08

**Authors:** Christopher Limb, Alex Fowler, Buket Gundogan, Kiron Koshy, Riaz Agha

**Affiliations:** a St Helier Hospital, Surrey; b Guy's and St Thomas' NHS Foundation Trust; c University College London Medical School; d Brighton and Sussex University Hospitals, London, UK

**Keywords:** Audit, Quality improvement project, QIP, Clinical governance, Service improvement, Project design, Design, Leadership and management

## Abstract

Audits and quality improvement projects are vital aspects of clinical governance and continual service improvement in medicine. In this article we describe the process of clinical audit and quality improvement project. Guidance is also provided on how to design an effective audit and bypass barriers encountered during the process.

## What is a clinical audit?

An audit assesses if a certain aspect of health care is attaining a recognized standard. This lets care providers and patients know where their service is doing well, and where there could be improvements. The aim is to achieve quality improvement and improve outcomes for patients.

Audits are a quality improvement measure and one of the 7 pillars of clinical governance. It allows organizations to continually work toward improving quality of care by showing them where they are falling short, allows them to implement improvements, and reaudit or close the audit cycle to see if beneficial change has taken place.

Clinical audits are a cycle with several steps:

 

**Figure:**
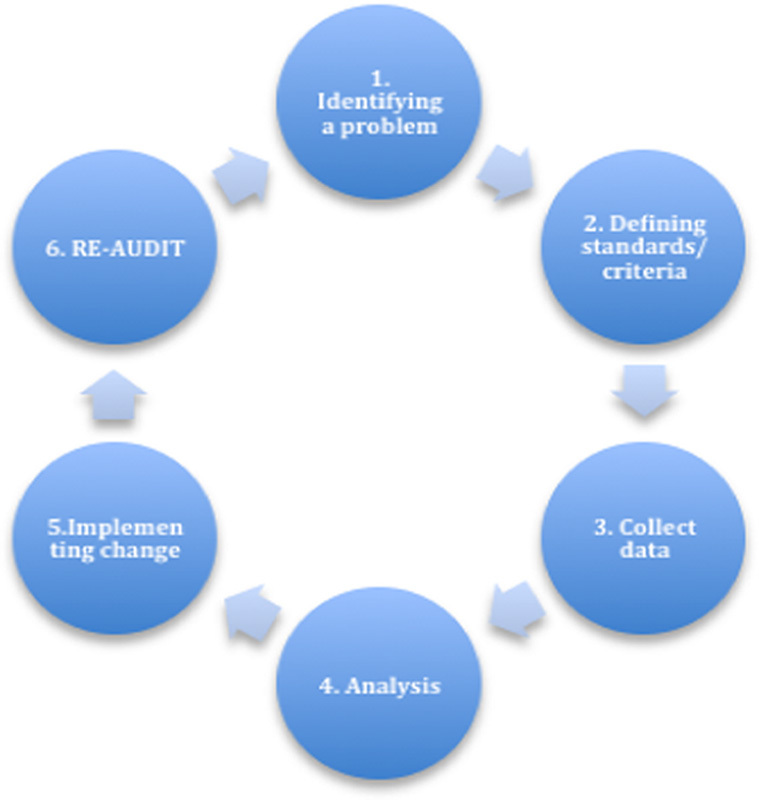

1. Identifying a problem, for example, patients waiting too long in accident and emergency (A&E).
2. Defining standards/criteria, for example, guidelines recommend that 95% of patients should wait <4 hours.
3. Collect data, for example, record over a week how long patients wait (determining that 92% wait for <4 h).
4. Analysis, for example, 92% versus 95% target, areas for improvement.
5. Implementing change, for example, action plan to help reach target.
6. Reaudit, for example, do step 1–5 again.

## What is quality improvement?

Quality improvement (QI) aims to improve the patient experience. Although audit is often more clinically orientated, QI can focus on more holistic issues, for example, the availability of hot drinks in A&E. QI can be done using the plan, do, study, act (PDSA) framework. PDSA cycles are iterative and have short time spans allowing improvement to be incorporated quickly^1^.

 

**Figure:**
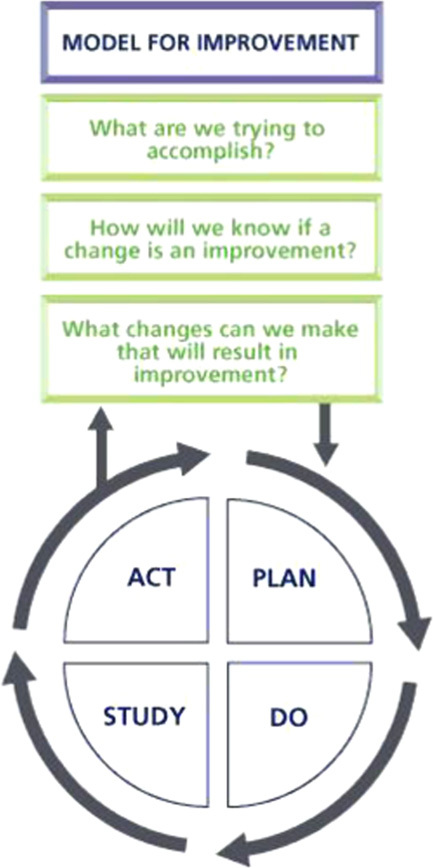


## How do audit and QI differ?

Audit and QI projects (QIPs) are essentially the same thing, they both look at how health care standards are, and aim to improve them—it’s just that audits have a more formal standard to measure against and also tend to have a longer time period, for example, done once every few months. PDSA cycles used in QI can be weekly or even daily.

## Why get involved in audit and QI?

- Because you will help to improve patient care.
- It’s a great way to show interest in a certain field.
- You’ll learn many transferable skills, for example, teamwork, time management.
- They can be presented at conferences, or written up for publishing.
- Completed audits are extra CV points for specialist training.
- As a doctor, it may form part of your annual appraisal and assessments!
- It is a mandatory requirement for UK junior doctors to progress in their training.

## How to get involved

There are 2 ways of getting involved in audit/QI:

1. Joining an already organized audit.
2. Setting up an audit yourself.

## Joining an organized audit/QIP

These audits are already planned, and need people to do the data collection (step 3). These may be good for people who haven’t done an audit before, or if they would like to get involved with one that is multicenter.

- Ask doctors if they have any audits that you could help with.
- In the United Kingdom, trainee research collaboratives such as STARSurg run multicenter national audits. Here there is a protocol, and your role is to do the data collection at your site. The data is often processed centrally.
- University societies such as Acamedics have many audits available for students to get involved with that have already been planned by doctors.
  - PROS—good introduction to auditing, less time commitment.
  - CONS—less flexibility, fewer skills learnt, fewer potential perks—for example, conferences/CV points.

## Planning your own audit/QIP

In planning your own audit, you have free reign to pick something that you’re interested in.

- Use the SMART criteria when thinking about planning:
  - Specific—choose a certain area that you’re interested in, and don’t keep it too broad. For step 1, your problem should be 1 short sentence.
  - Measurable—there should be something you can audit against, a local or a national target. (Choosing a national target may be easier to present at conference/get published.) Be sure to define your standards/criteria well, and that these are objective, for example, “Temp >38 degrees” not “fever.”
  - Achievable—limit yourself to 1 or 2 outcomes. Ensure the data are easily collectable, and that you have all the relevant access before you start, for example, computer logins. Are you at the hospital long enough to do several reaudits?
  - Realistic—are you the right person for this audit? It is easier for a student to assess 4 hour waits in A&E than rates of a certain technique being used in theater.
  - Timely—choosing something that can be done quickly will keep you motivated, and give you more opportunity to reaudit.
- Remember to get permission from the audit lead of the department and register it with the audit department.
- Engaging all stakeholders or people whom your work will impact (nurses, physios, pharmacists, etc., in addition to doctors) early on is vital, and gaining their feedback will be useful.
- Involving seniors may make step 5—implementing change easier, and will prevent you from doing an audit that is already being done.
- Reauditing is a MUST. Doing this 3 times will achieve maximum marks in most training applications. An audit that has a high turnover of patients and an easy change to implement is easier to reaudit than one with lower numbers of patients and a more difficult intervention.
- Present your results at meetings/conferences, for example, International Conference on Quality and Safety and RCS Edinburgh Audit Symposium, and try and get them published. Reporting should follow the Squire Guidelines^2^.
  - PROS—more flexibility, more CV points.
  - CONS—bigger time commitment, more admin/planning.

## Summary

Audits and quality improvement are a good way to learn more about a certain field, show interest, and learn new skills.

- Good planning is essential.
- Always reaudit!
- Try and present your results locally or nationally, or even submit for publication.
- Report using SQUIRE Guidelines.
- Publish them—for example the *Annals of Medicine and Surgery* (http://www.annalsjournal.com) is indexed in PubMed and publishes audits.
